# Predicting protein complex in protein interaction network - a supervised learning based method

**DOI:** 10.1186/1752-0509-8-S3-S4

**Published:** 2014-10-22

**Authors:** Feng Ying Yu, Zhi Hao Yang, Nan Tang, Hong Fei Lin, Jian Wang, Zhi Wei Yang

**Affiliations:** 1 College of Computer Science and Technology, Dalian University of Technology, Dalian, China; 2 Department of Ultrasound, Oil Field Hospital of Daqing, Heilongjiang, China

**Keywords:** Protein-protein interaction network, Protein complexes, Gene Ontology, Supervised learning

## Abstract

**Background:**

Protein complexes are important for understanding principles of cellular organization and function. High-throughput experimental techniques have produced a large amount of protein interactions, making it possible to predict protein complexes from protein -protein interaction networks. However, most of current methods are unsupervised learning based methods which can't utilize the information of the large amount of available known complexes.

**Methods:**

We present a supervised learning-based method for predicting protein complexes in protein - protein interaction networks. The method extracts rich features from both the unweighted and weighted networks to train a Regression model, which is then used for the cliques filtering, growth, and candidate complex filtering. The model utilizes additional "uncertainty" samples and, therefore, is more discriminative when used in the complex detection algorithm. In addition, our method uses the maximal cliques found by the Cliques algorithm as the initial cliques, which has been proven to be more effective than the method of expanding from the seeding proteins used in other methods.

**Results:**

The experimental results on several PIN datasets show that in most cases the performance of our method are superior to comparable state-of-the-art protein complex detection techniques.

**Conclusions:**

The results demonstrate the several advantages of our method over other state-of-the-art techniques. Firstly, our method is a supervised learning-based method that can make full use of the information of the available known complexes instead of being only based on the topological structure of the PIN. That also means, if more training samples are provided, our method can achieve better performance than those unsupervised methods. Secondly, we design the rich feature set to describe the properties of the known complexes, which includes not only the features from the unweighted network, but also those from the weighted network built based on the Gene Ontology information. Thirdly, our Regression model utilizes additional "uncertainty" samples and, therefore, becomes more discriminative, whose effectiveness for the complex detection is indicated by our experimental results.

## Background

Most proteins form complexes to accomplish their biological functions [1,2]. Protein complexes are important for understanding principles of cellular organization and function. While there are a number of ways to detect protein complexes experimentally, Tandem Affinity Purification (TAP) with mass spectrometry [3] is the preferred experimental detection method used by many research groups. However, there are several limitations to this method [4]. For example, its multiple washing and purification steps tend to eliminate transient low affinity protein complexes. Also, the tag proteins used in the experiments may interfere with the protein complex formation. Gavin et al. [1] have shown that TAP-MS only captures limited known yeast protein complex subunits. Furthermore, in TAP-MS the subcellular location of complexes is lost due to the *in vitro *purification of whole-cell lysates [5]. This means that time-consuming preparation of subcellular fractionated lysates may be needed for a less-studied cellular process in order to employ subcellular localization information to validate the experimental results and detect false negatives or false positives. Due to these experimental limitations, alternative computational approaches for detecting the complexes are thus useful complements to the experimental methods for detecting protein complexes [6].

In the post-genome era, high-throughput experimental techniques have produced a large amount of protein interactions, making it possible to predict protein complexes from the protein interaction networks (PIN). Automatic complex identification approaches are increasingly proposed to extract the set of proteins from the PIN as complexes.

The PIN can be described as a graph, the nodes of which correspond to the proteins and the edges correspond to the interactions; thus, the complex detection is realized by finding the subgraphs from PIN. Since the proteins in the same complex are highly interactive with each other, the protein complexes generally correspond to the dense subgraphs in the PIN [7]. Therefore, the proposed complex detection methods can be roughly divided into four categories. (1) Agglomerative model, in which every single node or some subgraph forms a cluster at the beginning stage and clusters are allowed to merge and grow under certain constraints. For example, the MCODE method is based on growing seeds selected by weight [8]. Similarly, the DPClus method expands clusters starting from seeded vertices [9]. (2) Clique finding methods. The CFinder system finds functional modules in PIN by detecting the k-clique percolation clusters using a Clique Percolation Method [10]. CMC is also a clique based method that uses a protein-protein interaction iteration method to update the network [11]. The ClusterONE method initiates from a single seed vertex before a greedy growth procedure begins to add or remove vertices in order to find groups with high cohesiveness [12]. (3) Traditional graph clustering methods based on a premise that PIN can be described as a graph, thus the algorithm can also be applied to detect dense clusters as protein complexes. The Markov clustering method (MCL) simulates random walks within graphs and thus partitions the PPI network into many non-overlapping dense clusters [13].(4) Complex detection methods based on the core-attachment architecture developed by Gavin et al., who demonstrated that protein complex had the architecture of core-attachment [2]. An example of such methods is COACH that selects some subgraph as the core structure first, and then adds the attachment to the core to construct a complex [14].

However, most of above methods are unsupervised learning methods, which predict the protein complexes based on the pre-defined rules. Although these unsupervised learning methods have the superiority of solving the problem without annotation and training process, they can not make full use of the information of available known complexes. In the research field of protein complexes, numerous true complexes have been provided, which can be used as the prior knowledge of the supervised learning method. Qi et al. first imported the supervised learning method into the complex detection. By using graph topological patterns and biological properties as features, they trained a probabilistic Bayesian network (BN) model score subgraphs in the protein interaction graph and identify new complexes [15]. Shi et al. proposed a semi-supervised prediction model with neural network and their results shows that integrating biological features and topological features to represent protein complexes is more meaningful than using dense subgraphs [16]. Chen et al. analyzed the graph properties and biological properties of protein complexes and constructed a prediction model using the filtered features [17]. However, their method only determines whether a candidate protein complex is a true complex and doesn't deal with the construction of the candidate protein complexes from the PIN. Qiu et al. developed multiple kernels from heterogeneous data sources and combined them in an SVM classifier to predict co-complexed protein pairs [18]. Like Chen et al., their method also doesn't deal with the construction of the candidate protein complexes from the PIN although the co-complexed protein pairs it predicts can extend known MIPS complexes and identify maximal cliques as candidate protein complexes.

In this paper, we present a supervised learning based method to discover the complexes in the PIN by learning from true complexes. Compared with other supervised learning based methods (e.g. Qi et al.[15] and Shi et al.[16]), our method introduces some new features from the weighted network: the density, the mean and maximum degrees of the weighted network, which prove to be quite effective for the performance improvement. In addition, our method uses the three categories training set for the first time. Since the more samples and additional categories provide more information for the regression model training, the learned model becomes more discriminative. Finally, our method uses the maximal cliques found by the Cliques algorithm [19] as the initial cliques, which has been proven to be more effective than the method of expanding from the seeding proteins used in other methods.

## Methods

### Complex detection algorithm

The aim of complex detection is to discover subgraphs representing the predicted protein complexes from the PIN. We propose a supervised learning-based method including four steps as shown in Table 1. The inputs are an unweighted network, a weighted network and a training set. The unweighted and weighted networks are originally constructed from the DIP database (the Database of Interacting Proteins [20]), which contains 4928 proteins and 17201 interactions and then the interactions with GO similarities less than 0.9 are regarded as false positive interactions and deleted from the PIN as will be discussed in the following section. The size of the training set is of great importance for the supervised learning-based method. However, currently it is difficult to obtain sufficient number of positive training samples in complex detection field. Thus, in order to achieve more training samples, we used 422 complexes which are predicted by the COACH method but do not match the true complexes in the benchmark. Since the COACH method is a state-of-the-art complex detection method, its predicted result that doesn't match a true complex could still be a true complex. We assigned them "uncertainty" status denoting that their potential of being true complexes is superior to the negative samples and inferior to the positive ones. Consequently, we constructed three different training sample categories: 668 true complexes from some available PPI databases are used as the positive samples, 422 complexes predicted by the COACH method as the intermediate samples, and 2004 subgraphs obtained by randomly selecting nodes as the negative samples. The more samples and additional categories provide more information for the learning model to be more discriminative.

**Table 1:** Protein complex detection algorithm

**Input **: an unweighted network, a weighted network built via GO annotation and a training set Complex detection process: **Step 1**: construct the feature vector space for the complexes in the training set from the unweighted and weighted PIN networks and train the Regression model **Step 2**: find maximal cliques in the PIN by the Cliques algorithm -rank the clique set C={C_1_, C_2_, ..., C_n_} in descending order of the scores given by the Regression model -for each clique C_i_, check all the cliques (denoted as C_j_) with lower scores, if C_i_∩C_j _> threshold, then remove C_j_. -output: the updated clique set **Step 3**: grow the cliques -for each clique C_i_, the set of its neighbors is denoted as N(C_i_), do update operation as follows: -check all the nodes in N(C_i_) -select v_i_∈N(C_i_), which makes v_i_∪C_i _achieve higher score given by the Regression model -update C_i_= v_i_∪C_i_, N(C_i_) = N(C_i_) - v_i_ -repeat the update operation until there is no node v_j _in N(C_i_) that leads to score(v_j_∪C_i_) > score(C_i_) -output: the candidate complex set C = {C_1_, C_2_, ..., C_n_} **Step 4**: filter the candidate complexes -rank the candidate complexes in descending order of the score given by the Regression model -for each candidate complex C_i_, check all the candidates C_j _with lower scores -if overlap (C_i_, C_j_) > merg_thred if score(C_i_∪C_j_) > score(C_i_) do merge operation: update C_i _= C_i_∪C_j_ else do remove operation: remove C_j _from the candidate set **output**: the predicted complex set

In the first step, the feature vectors are generated for the complexes in the training set from the unweighted and weighted PIN networks based on the features which will be discussed in the later section of *Complex feature selection*. It should be noted that all the features are extracted from the true protein complexes when they are in the PIN (i.e. the true protein complexes are the (unweighted or weighted) subgraphs in the whole (unweighted or weighted) protein interaction network. The Regression model is subsequently trained by solving the optimization problem by gradient descent.

In the second step, the Cliques algorithm is used to find maximal cliques in the PIN [19]. Although enumerating all maximal cliques is NP-hard, this does not pose a problem in PPI networks because PPI networks are usually sparse [11]. The Cliques algorithm uses a depth-first search strategy to enumerate all maximal cliques, and it can effectively prune non-maximal cliques during the enumeration process. In our experiments, we explored two different minimal sizes of the cliques on the performance: the sizes greater than or equal to 3 and 4 (denoted as clique_size ≥ 3 and clique_size ≥ 4 respectively). Furthermore, because of the high density of the PIN, the cliques may have high node overlapping rate. For example, two cliques with four nodes may have three nodes in common. Therefore, the cliques are filtered as follows: the set of cliques is ranked in descending order of their scores given by the Regression model, denoted as {C_1_, C_2_, ..., C_k_}; for each clique C_i _(i = 1, 2, ..., k), whether the number of common nodes of C_i _and the clique C_j _(j = i + 1, ..., k and C_j _has a lower score than C_i_) is larger than or equal to the threshold (set to 2 and 3 for clique_size ≥ 3 and clique_size ≥ 4 respectively) is checked. If so, the clique with the lower score is removed.

In the third step, the growing operation is performed on each clique obtained in the previous step. For a clique C_i_, the set of its neighbors is denoted as N(C_i_) and, for each node v_i _in N(C_i_), it is checked if its addition to C_i _makes the new subgraph { C_i_∪v_i_} obtain higher score given by the Regression model. The operation is repeated until no node introduction leads to higher score of the new subgraph. Thus, after the growing operation, the cliques constitute a set of candidate complexes.

The candidate complexes may still have a high overlapping rate since they also may have some neighbor nodes in common. Therefore, in the fourth step, similar filtering operation as in the second step is performed. For two candidate complexes, C_1 _= {p_1_, p_2_, ..., p_m_} and C_2 _= {q_1_, q_2_, ..., q_n_}, their overlapping rate is calculated as follows:

(1) $$overlap\left(C_{1},C_{2}\right)=\frac{\left|C_{1}\cap C_{2}\right|}{\left|C_{1}\cup C_{2}\right|}$$

The merging threshold (denoted as merg_thred) is set to a value between 0 and 1. The merging operation is performed as follows: first, the candidate complexes are ranked in descending order of their scores given by the Regression model; then, for each candidate complex C_i_, its overlapping rate with all the candidates C_j _with lower scores are calculated. If the overlapping rate is higher than the merg_thred, the merging operation is performed if the score of their union is higher than that of C_i _itself. Otherwise, the complex C_j _is removed.

### Weighted network construction

The PIN can be modeled as a simple graph G = (V, E), in which a node element in node set V represents a protein and an edge element in edge set E represents an interaction between two distinct proteins. In our method, a weighted graph is introduced to represent PIN as G = (V, w(E)), where w(E) represents the weighted interaction. In this way, we extract the complex features based on two different networks--an unweighted and a weighted network.

Protein interaction data produced by high-throughput experiments are often associated with high false positive and false negative rates due to the limitations of the associated experimental techniques and the dynamic nature of protein interaction maps. Therefore, the complex features extracted from the unweighted network are insufficient for describing a complex. Gene Ontology (GO) provides a collection of well-defined biological terms--known as GO terms--spanning biological processes, molecular functions and cellular components. Here, based on the method presented in [21], we use GO annotation from SGD [22] to estimate the similarity between proteins, and then use it as the weight of network.

In our method, the semantic similarity between two proteins is calculated based on the annotation size of the GO term (which is defined as the number of annotated proteins on the GO term) on which both proteins are annotated. According to the transitivity property of GO annotation, if a protein *p *is annotated on a GO term *gi*, it is also annotated on the GO terms on the path from *gi *to the root GO term in the GO structure. Thus, the proportion of the annotation size of a GO term to the total number of annotated proteins can quantify the specificity of the GO term. If two proteins are annotated on a more specific GO term and have more common GO terms, then they are functionally more similar. We define the semantic similarity *sim*(*p, q*) between two proteins *p *and *q *as follows:

(2) $$sim\left(p,q\right)=-|C\left(p,q\right)|\times \text{log}\left(\frac{\text{min}|Si\left(p,q\right)|}{Smax}\right)$$

*C*(*p,q*) denotes the set of the GO terms whose annotation includes *p *and *q*. If both *p *and *q *are annotated on *n *different GO terms, *Si*(*p, q*) (1≤*i*≤*n*) denotes a set of annotated proteins on the GO term *gi *whose annotation includes *p *and *q. Smax *is the maximum size of annotation among all GO terms in a directed acyclic graph (DAG) structure. The proportion of the annotation size of a GO term (*Si*(*p,q*)) to the total number of annotated proteins (*Smax*) can quantify the specificity of the GO term. If *p *and *q *are annotated on a more specific GO term and more common GO terms than *p *and *l *(another protein), then *p *is semantically more similar to *q *than *l*. In addition, the graph topology is also introduced into the weight calculation. For an input graph *G *= (*V, E*), we assign the topological weight of an edge [*u, v*] to be the number of neighbors shared by the vertices u and v (which represent proteins *p *and *q *respectively). Then the sum of *sim *(*p, q*) and topological weight is assigned to the edge between *u *and *v*.

In our experiments, if proteins are not annotated by the GO terms, 0 is used as their interaction weight and the interactions with GO similarities less than 0.9 are regarded as false positive interactions and deleted from the PIN.

### Complex feature selection

Extracting appropriate features for the subgraphs representing complexes is related to the problem of measuring the similarity between complex subgraphs. We designed the following features to describe a complex subgraph in the PPI network. Some features are extracted from the unweighted network and other features from the weighted network.

1. Graph density: The graph density has been used in many complex detection methods, and it has been proven to be an important feature for complex detection [8]. Let G = (V, E) be an unweighted graph, with |V| vertices and |E| edges. Suppose |E|_m _=|V|(|V|-1)/2 is the theoretical maximum number of possible edges in G, and the unweighted graph density is defined as the ratio of |E| and |E|_m_. For the weighted graph, the weight of the edge <u, v> is given by G = (V, w(E)), w(u, v). Thus, the density of the weighted graph is defined as follows:

(3) $$\text{densit}{\text{y}}_{\text{w}}\left(G\right)=\frac{\sum_{u\in \text{V},v\in \text{V}}w\left(u,v\right)}{|\text{V}|\cdot \left(|\text{V}|-1\right)}$$

2. Degree statistics: Degree is defined as the number of neighbors of a node in unweighted graph that describes the connection between the nodes. For the unweighted graph, the mean and medium degrees are chosen as the node degree feature. In the weighted graph, a degree is defined as the sum of the weights between the node and its neighbors and the mean and maximum degrees are chosen as the node degree features.

3. Edge weight statistics: Similar to the node degree, the edge weight is another important measure of the weighted network as it describes the feature of the edge. The mean of all weights is chosen as the edge weight statistics feature.

4. Clustering coefficient: Clustering coefficient reflects the neighbors of the nodes that can be used to describe the modularity of the graph. Let G = (V, E) be a complex graph with V = {v_1_, v_2_,..., v_n_}(n is the number of nodes). For each node v_i_, the set of its neighbors is denoted as V_i_' = {v_i1_, v_i2_, ..., v_ik_} and let N_i _= (V_i_', E_i_) be an induced graph of G. Define C_i _= 2|E_i_|/k(k-1) (if k ≤ 1, C _i_= 0), where k denotes the number of nodes in V_i_'. The mean of {C_1_, ..., C_n_} is chosen as the clustering coefficient feature [23].

5. Topological change: For a weighted graph, topological change features are gained by measuring the topological changes when different weight cutoffs are applied to the graph (ranging from 1 to 8). Let G_i _= (V, E_i_) (*i *= 1, ..., 8) be the graphs in which only the edges with the weights higher than *i *remained, that is, E_i _= {e|w(e) >*i*}. Topological changes are measured as T_i _= (|E_i_|-|E_i+1_|)/|E_i_| (i = 1, ..., 7. If |E_i_| = 0, let T_i _= 0). In our feature set, T_i _(*i *= 2, ..., 7) are chosen as the topological change features [17] which measure the distribution of the edge degrees in the weighted network.

The five groups of features discussed above are used in our experiments for describing the complexes from different perspectives (as shown in Table 2). Four features are based on the unweighted network and six features are based on the weighted network.

**Table 2:** Feature distribution

Features of the unweighted network			Features of the weighted network		
**Group** **ID**	**Feature**	**Number of features**	**Group** **ID**	**Feature**	**Number of features**

1	Graph density	1	1	Graph density	1
2	Degree statistics	2	2	Degree statistics	2
4	Clustering coefficient	1	3	Edge weight statistics	1
-	-	-	5	Topological change	2

### Regression model

In our method, the Regression model is introduced to evaluate the possibility a subgraph is a true complex. Regression analysis is a statistical method used to model and analyze several features [24]. The goal of regression is to summarize observed data as simply, usefully and elegantly as possible [25]. In the context of the complex detection problem, a model that can evaluate the possibility a subgraph is a true complex is required. In our method, the regression analysis is used to model the complex detection problem, as it can train a model of the multiple features by analyzing the training set.

We model the problem evaluating the possibility a subgraph is a true complex as a linear regression function, $\text{f}\left(x\right)=\omega^{T}\cdot x$, where *f(x) *is the linear function of features and *ω^T ^*is the weight vector of the dimension corresponding to the number of the features. The linear least square approach is used to obtain the regression model, and the least square function is defined as follows:

(4) $$L=\overset{N}{\underset{i=1}{\sum }}{\left(y_{i}-f\left(x_{i}\right)\right)}^{2}$$

where *i *is the training sample, *N *the number of the samples, *y_i _*the annotation of sample *i *(in our model yi∈{0,1,2}. For the negative samples, intermediate samples and positive samples, *y_i _* is set to 0, 1 and 2 respectively), *x_i _* the feature vector, and $f\left(x_{i}\right)$ the score of the sample *i *. This approach leads to an optimization problem, whereby, when the least square function obtains the least value, the model is optimal.

We solve the optimization problem by the gradient descent algorithm, which is an iterative algorithm where, within each step, the gradient of the objective function is calculated, and then the negative direction of gradient is used to search the next step by multiplying the step-size. The gradient of the object with respect to parameter can be calculated as follows:

(5) $$\text{Δ}\omega_{j}=2\overset{N}{\underset{i=1}{\sum }}\left(y_{i}-f\left(x_{i}\right)\right)\cdot x_{ij}$$

where *ω_j _* is the weight of *j*th dimension, and *ω * is updated by ω←ω-η⋅Δω, and the learning rate *η * can be set to a small positive value.

### Datasets

Our method was tested on the DIP database, which has been widely used in complex detection field, so that our result is comparative with others. DIP contains 4928 proteins and 17201 interactions. We built the weighted network by calculating the GO similarity of the proteins as discussed in the previous section. 6120 interactions with GO similarities less than 0.9 were deleted since the lower GO similarity indicates that two proteins have less common functional annotations and their interaction is more likely to be a false one.

**Figure 1:**
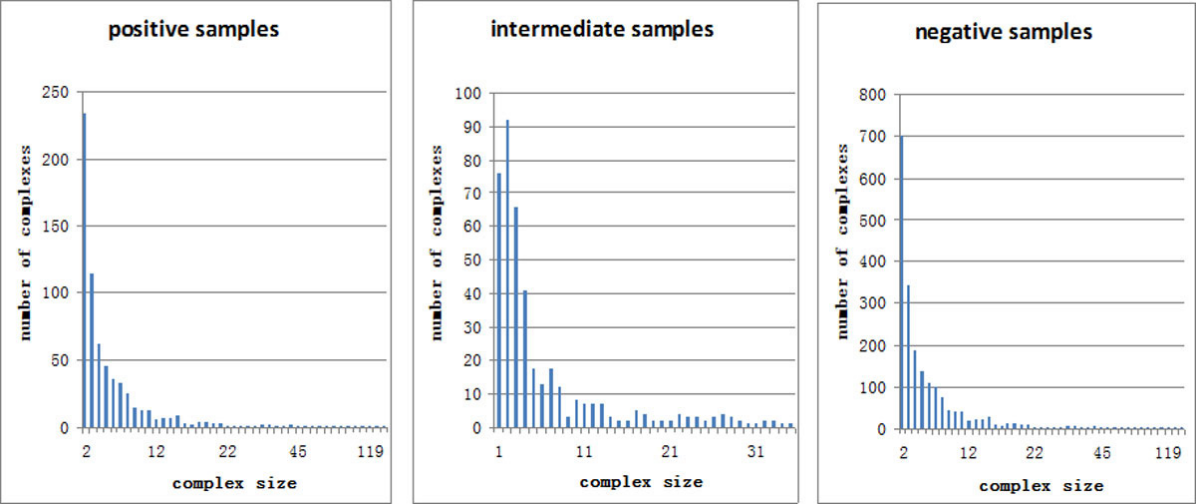
**Size distribution of the positive, intermediate and negative samples**. Horizontal axis denotes the complex size and vertical axis denotes the number of complex samples with certain sizes.

Our training set includes 668 positive samples, 422 intermediate samples and 2004 negative samples. The positive samples are obtained from four sources: (I) MIPS [26], (II) Aloy et al., [27], (III) SGD database [22] and (IV) TAP06 [2]. Moreover, as the extant research shows that most of the complexes include more than one protein [28], we choose the complexes which at least have two different proteins as the true complexes. The intermediate samples are 422 complexes predicted by the COACH method, and 2004 subgraphs obtained by randomly selecting nodes are used as the negative samples. The size distribution of the positive sample set follows a power law, so do the intermediate and negative sample sets as shown in Figure 1. After a Regression model is trained with the training set, our complex detection algorithm is performed on the DIP PIN. Then the detected complexes are evaluated using the metrics to be introduced in the following section.

### Evaluation metrics

The neighborhood affinity score (NA (A, B)) [29] is used as a measure to evaluate the similarity of two given clusters A and B, and is defined as follows:

(6) $$NA\left(A,B\right)=\frac{|VA\cap VB{|}^{2}}{\left|VA|\times |VB\right|}$$

The neighborhood affinity score between a predicted complex p and a true complex b, NA (p, b), is used to determine whether they match. If NA (p, b) ≥ *ω *, they are considered to be matching (*ω * is usually set as 0.25). Let P and B be the sets of the predicted and true complexes in the benchmark respectively, *Ncp *be the number of the correct predictions that match at least a true complex and *Ncb *be the number of the true complexes that match at least one predicted complex, the precision and recall are defined as follows:

(7) $$precision=\frac{N_{cp}}{\left|P\right|},recall=\frac{N_{cb}}{\left|B\right|}$$

The F-measure (the harmonic mean of precision and recall and defined as (2PR) / (P + R) where P denotes precision and R recall) can be used to evaluate the overall performance, which is a popular metric in the performance evaluation of complex detection methods.

Recently, the sensitivity (*Sn*), positive predictive value (*PPV*), accuracy (*Acc*) and *P-value *have also been proposed to evaluate the performance of complex detection methods [29]. Given *n *benchmark complexes and *m *predicted complexes, let *T_ij _*be the number of proteins in common between *i*th benchmark complex and *j*th predicted complex, *Sn *and *PPV *are then defined as follows:

(8) $$Sn=\frac{\sum_{i=1}^{n}\underset{j}{\text{max}}\left\{T_{ij}\right\}}{\sum_{i=1}^{n}N_{i}}$$

(9) $$PPV=\frac{\sum_{j=1}^{m}\underset{i}{\text{max}}\left\{T_{ij}\right\}}{\sum_{i=1}^{m}T_{.j}}$$

where *N_i _*is the number of proteins in the ith benchmark complex, and *T_.j _*is defined as:

(10) $$T_{.j}=\sum_{i=1}^{n}T_{ij}$$

Generally, a high *Sn *value indicates that the prediction has a good coverage of the proteins in the true complexes, whereas a high *PPV *value indicates that the predicted complexes are likely to be true complexes. As a summary metric, the accuracy of a prediction, *Acc*, can then be defined as the geometric average of *Sn *and *PPV*:

(11) $$Acc=\sqrt{Sn\times PPV}$$

These metrics are by no means absolute measures--they all have their own limitations, *Sn, PPV *and *Acc *in particular. For example, if a method predicts a giant complex that covers many proteins in the known true complex set, this method will yield a very high *Sn *score. Similarly, *PPV *value cannot evaluate overlapping clusters reliably. Here is a case in point: if the known gold standard MIPS complex set is taken to match with itself, then the resulting *PPV *value is 0.772 instead of 1, although the precision and recall are both correctly calculated as 1. In such cases, the *Acc *score, as the geometric average of *Sn *and *PPV*, will not make sense either. Therefore, in the performance comparison, the F-measure is used as the main metric, and the *Acc *is only used as an auxiliary one.

*P-value *refers to the statistical significance of the occurrence of a predicted protein complex with respect to a given functional annotation, which is computed by the following hypergeometric distribution:

(12) $$P-value=1-\overset{k-1}{\underset{i=0}{\sum }}\frac{\left(\begin{array}{c} \left|F\right|\\ i \end{array}\right)\left(\begin{array}{c} \left|V|-|F\right|\\ |C|-i \end{array}\right)}{\left(\begin{array}{c} \left|V\right|\\ \left|C\right| \end{array}\right)}$$

where a predicted complex *C *contains *k *proteins in the functional group *F *and the entire PPI network contains |*V*| proteins. The functional homogeneity of a predicted complex is the smallest p-value over all the possible functional groups. A predicted complex with a low *p-value *indicates that it is enriched by proteins from the same function group and it is thus likely to be a true protein complex.

## Results and discussions

The effect of different parameters (e.g. Regression model iteration time, two or three category training set, clique_size and merg_thred) and feature sets on performance, the performance comparison with other methods and the statistical evaluation of the predicted protein complexes is discussed in this section.

It should be noted that in the experiments which will be discussed in the following two sections (*the effect of different parameters on performance *and *the effect of features set on performance*), we used the 668 positive samples as benchmark to evaluate our identified complexes which means the training and the testing data have overlap. In classical classification task, the problem will would affect the validity of the results. However, in our complex detection method, the problem is not so serious since the Regression model trained by the training data is not used directly to classify the candidate complexes to be true ones or not but to assign them scores used for the cliques filtering, growth, and candidate complex filtering as described in previous section. Nevertheless, to avoid the problem as possible as we can, when comparing our results with those of other methods we used a method (which will be introduced in the section *Performance comparison with other methods*) similar to the five-fold-cross-validation or different training and testing data. However, this five-fold-cross-validation method will lead to the significant increase of experiment time. For example, the number of experiments needed for Table 3 will increase from 144 (16*9) to 720 (16*9*5). Therefore, in the experiments introduced in, the following two sections we still used the 668 positive samples as benchmark to approximately evaluate the impacts of the parameters such as Regression model iteration time, two or three category training set, clique_size, merg_thred, and different feature sets on the performance while in the section *Performance comparison with other methods *(which compares our results with those of other methods) we used the five-fold-cross-validation method or different training and testing data.

**Table 3:** Performance comparison between different Regression models (clique_size ≥ 3)

	merg_thred								
**Model**	**0.1**	**0.2**	**0.3**	**0.4**	**0.5**	**0.6**	**0.7**	**0.8**	**0.9**

Model100	0.5244	0.5429	0.5467	0.5518	0.5628	0.5656	0.5791	0.5806	0.5719
Model200	0.5060	0.5380	0.5542	0.5525	0.5577	0.5586	0.5798	0.5819	0.5748
Model300	0.5070	0.5353	0.5451	0.5543	0.5637	0.5696	0.5887	0.5866	0.5783
Model400	0.5058	0.5414	0.5427	0.5578	0.5660	0.5730	0.5880	0.5904	0.5808
Model500	0.5115	0.5462	0.5465	0.5596	0.5710	0.5747	0.5896	**0.5910**	0.5783
Model600	0.4942	0.5368	0.5380	0.5460	0.5544	0.5667	0.5819	0.5831	0.5741
Model700	0.4995	0.5378	0.5417	0.5408	0.5498	0.5617	0.5713	0.5737	0.5692
Model800	0.5042	0.5331	0.5428	0.5402	0.5470	0.5544	0.5632	0.5676	0.5660
Model900	0.4962	0.5251	0.5338	0.5320	0.5355	0.5513	0.5592	0.5638	0.5627
Model1000	0.4966	0.5282	0.5339	0.5323	0.5322	0.5491	0.5540	0.5604	0.5598
Model1500	0.4936	0.5243	0.5273	0.5240	0.5278	0.5435	0.5563	0.5605	0.5597
Model2000	0.4944	0.5126	0.5185	0.5221	0.5262	0.5344	0.5487	0.5516	0.5513
Model2500	0.4813	0.5129	0.5183	0.5215	0.5239	0.5292	0.5418	0.5431	0.5426
Model3000	0.4849	0.5142	0.5210	0.5192	0.5233	0.5297	0.5406	0.5409	0.5401
Model3500	0.4749	0.5161	0.5247	0.5215	0.5231	0.5299	0.5391	0.5390	0.5379
Model4000	0.4789	0.5143	0.5238	0.5157	0.5200	0.5257	0.5368	0.5368	0.5357

### The Effect of different parameters on performance

**Figure 2:**
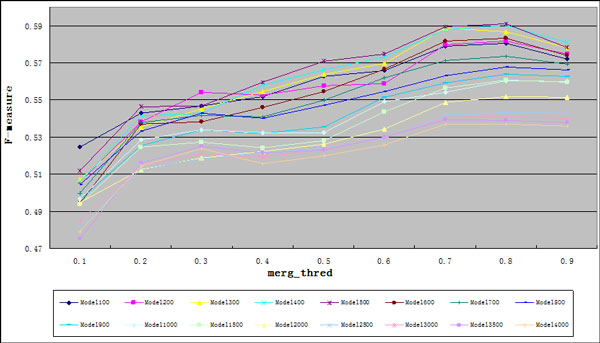
**F-measure curve of different models**. Horizontal axis denotes the merg_thred and vertical axis denotes the F-measure.

In our method, we imported the Regression model to evaluate the possibility a subgraph is a true complex. In Regression model, the regression square error is reduced as the time of iteration grows, and it will return different models with different iteration times. The performance comparison measured by F-measure between these different Regression models is made in Table 3. In the table, Model100 denotes the model with 100 iterations; Model200 denotes the model with 200 iterations, and so on. Among others, the Model500 achieves the highest F-measure of 0.5910 when merg_thred is 0.8 and clique_size ≥ 3. With the further increase of the iteration time, the F-measure begins to decrease. Through analyzing the result, we found that Model500 can achieve the higher precision than the models with more iteration time (e.g. Model4000) while they have almost the same recall. Figure 2 depicts the F-measure curves of different models, which shows that in most cases, with the increase of the merg_thred, the F-measure of each model keeps increasing and when the merg_thred is 0.7 or 0.8, it reach its peak value. Therefore, in the following experiments, the Regression model with 500 iterations and the merg_thred 0.8 are used.

**Figure 3:**
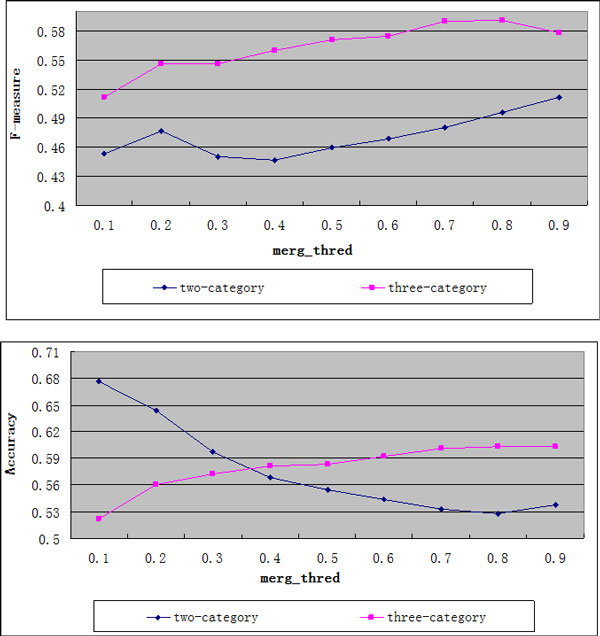
**F-measure and accuracy curves of two-category and three-category training sets**. Horizontal axis denotes the merg_thred and vertical axis denotes the F-measure and accuracy.

As mentioned in previous section, our Regression model is built with the three-category training set, which could improve the discrimination of the model. In order to prove its effectiveness, we made the performance comparison between the two-category and three-category training set with the clique_size ≥ 3. As can be seen from Figure 3, the F-measure and accuracy of using the three-category training set are much better than those of using the two-category training set when merg_thred is 0.8. Therefore, the three-category training set is used in our experiments.

In addition, we conducted the comparison experiments with different clique_sizes and merg_threds. Table 4 shows the performances when the clique _size ≥ 3, clique_size ≥ 4 and the merg_thred ranges from 0.1 to 0.9. The experimental results show that our method returns more complexes and achieves higher F-measure when the clique_size ≥ 3, as the lower clique_size will allow the Clique algorithm to find more cliques (more predicted complexes). On the contrary, when the clique_size ≥ 4, few predicted complexes are returned to match the true complexes. Therefore, we set clique_size ≥ 3 in our experiments. The advantage of clique_size ≥ 4 is that it returns fewer complexes with higher precision. For example, when merg_thred is 0.2, it returns 113 complexes and achieves the highest precision (0.7876). In addition, as the merg_thred grows from 0.1 to 0.9, the recall increases while the precision decreases. The reason is that, when the merg_thred increases, fewer merging operations are performed and our method can predict more complexes to achieve a high recall. However, the precision will deteriorate since more predicted complexes remain unmatched to any true complex. The F-measure achieves its highest value 0.5910 when the merg_thred is 0.8 (clique_size ≥ 3).

**Table 4:** Performance comparison of different clique_sizes and merg_threds

	Clique_size ≥ 4				Clique_size ≥ 3			
**merg_** **thred**	**Num**	**P**	**R**	**F**	**Num**	**P**	**R**	**F**

0.1	96	0.7813	0.2635	0.3941	212	0.6792	0.4102	0.5115
0.2	113	0.7876	0.2934	0.4275	286	0.6434	0.4746	0.5462
0.3	125	0.76	0.3009	0.4311	333	0.6186	0.4895	0.5465
0.4	142	0.7465	0.3084	0.4365	388	0.6237	0.5075	0.5596
0.5	171	0.7427	0.3129	0.4403	491	0.6273	0.5240	0.5710
0.6	203	0.7340	0.3159	0.4417	624	0.6298	0.5284	0.5747
0.7	248	0.7540	0.3189	0.4482	755	0.6530	0.5374	0.5896
0.8	303	0.7294	0.3234	0.4481	880	0.6477	0.5434	**0.5910**
0.9	368	0.6440	0.3249	0.4319	945	0.6180	0.5434	0.5783

Num denotes the number of the predicted complex, P the precision, R the recall and F the F-measure.

### The effect of different features set on performance

To evaluate the contribution of different feature sets to the performance, we conducted the experiments with different feature sets. The experimental results with three different feature sets--four unweighted features, seven weighted features, and all features - are showed in Table 5. The F-measures achieved with seven features from the weighted network are much better than those achieved with four features from the unweighted network, and almost as good as those using all features, which shows that the feature set from the weighted network is effective in improving the performance. The reason is that the weighted network feature set combines the GO information with the topological properties. In addition, the combination of the unweighted network feature set and weighted network feature set achieves an F-measure of 0.5910 (the merg_thred is 0.8), which indicates that the construction of our feature set is effective.

**Table 5:** Experimental results of three different feature sets

	Unweighted feature set				Weighted feature set				All feature set			
***merg_*** ***thred***	***Num***	**P**	**R**	**F**	***Num***	**P**	**R**	**F**	***Num***	**P**	**R**	**F**

0.1	169	0.5621	0.3009	0.3920	205	0.6537	0.4042	0.4995	212	0.6792	0.4102	0.5115
0.2	242	0.4876	0.3443	0.4036	266	0.6353	0.4536	0.5293	286	0.6434	0.4746	0.5462
0.3	309	0.4434	0.3757	0.4068	301	0.6146	0.4790	0.5384	333	0.6186	0.4895	0.5465
0.4	389	0.4216	0.4042	0.4127	350	0.62	0.4910	0.5480	388	0.6237	0.5075	0.5596
0.5	476	0.4202	0.4132	0.4166	443	0.6163	0.5090	0.5575	491	0.6273	0.5240	0.5710
0.6	576	0.4184	0.4266	0.4225	572	0.6189	0.5165	0.5631	624	0.6298	0.5284	0.5747
0.7	703	0.4296	0.4371	0.4333	717	0.6374	0.5329	0.5805	755	0.6530	0.5374	0.5896
0.8	815	0.4331	0.4431	**0.4381**	834	0.6319	0.5389	**0.5817**	880	0.6477	0.5434	**0.5910**
0.9	910	0.4286	0.4446	0.4364	930	0.6108	0.5404	0.5734	945	0.6180	0.5434	0.5783

We also analyzed the contribution of individual features to the performance. Table 6 shows the rank lists of the features achieved with two different standards. The Regression model assigns the features with different weights that reflect the importance of each feature, and the features are ranked by the weights in the descending order in Table 6 (the column 2 and 3 show the feature names and their weights).

**Table 6:** Two feature rank lists

Rank	Feature	Weight	Feature	F
1	Mean edge weight of the weighted network	0.1529	***Density of the weighted network***	0.5697
2	Density of unweighted network	0.0964	Mean edge weight of the weighted network	0.5709
3	***Density of the weighted network***	0.0243	***Mean degree of the weighted network***	0.5768
4	Mean of the unweighted clustering coefficient	0.0198	***Maximum degree of the weighted network***	0.5773
5	Topological change 7	0.0051	Density of the unweighted network	0.5884
6	Mean degree of the unweighted network	0.0047	Mean degree of the unweighted network	0.5884
7	Topological change 5	0.0044	Medium degree of the unweighted network	0.5884
8	**Maximum degree of **the **weighted network**	0.0040	Topological change 7	0.5889
9	Medium degree of the unweighted network	0.0027	Mean of the unweighted clustering coefficient	0.5896
10	***Mean degree of the weighted network***	0.0008	Topological change 5	0.5905

Moreover, the experiments were also conducted to verify the performance our complex detection method could achieve when each feature was removed. If the performance declines more sharply when a feature is removed, the feature is deemed more important. In this way, the features are ranked by the F-measure of each one-feature-removed experiment in the ascending order in Table 6 (the column 4 and 5 show the ranked feature names and their F-measures). It should be noted that topological change features help to enhance the performance only when *i *= 5 and 7, and, therefore, other topological change features are removed in our feature set.

In accordance with the results in Table 5 the features from the weighted network play a more important role in the feature set than those from the unweighted network. Among others, the mean edge weight and density features of the weighted network rank among top 3 in both lists. This is also consistent with the idea of the previous complex detection algorithms based on detecting density subgraph.

Compared with the other supervised learning-based methods of Qi et al. and Shi et al., our method introduces some new features from the weighted network (in bold and itlic in Table 6): the density, the mean and maximum degrees of the weighted network. Our experiment shows that these features are quite effective in complex detection: They totally contribute to a performance improvement of 2.6 percentage points in F-measure (from 0.5650 to 0.5910).

In order to prove the effectiveness of our Regression model, the comparative experiments between the Regression and the equal weight model that assigns all the features with the same weight were conducted. The results are shown in Table 7 from which it can be seen that the F-measures of the Regression model are superior to those of the equal weight model at different merg_threds. When the merg_thred is 0.8, the Regression model achieves an F-measure of 0.5910 which is significantly better than that of the equal weight model (0.4366). This indicates that the Regression model is effective in assigning appropriate weights to different features and, therefore, improving the performance.

**Table 7:** Performance comparison between the Equal Weight (EW) and Regression models (RM)

merg_ thred	model	Num	Ncp	P	Ncb	R	F	Sn	PPV	Acc
0.1	EW	167	94	0.5629	182	0.2725	0.3672	0.6075	0.6550	0.6308
	RM	212	144	0.6792	274	0.4102	0.5115	0.3473	0.7849	0.5221
0.2	EW	227	109	0.4802	217	0.3249	0.3875	0.6092	0.6635	0.6358
	RM	286	184	0.6434	317	0.4746	0.5462	0.4054	0.7749	0.5605
0.3	EW	289	127	0.4394	234	0.3503	0.3898	0.6190	0.6329	0.6259
	RM	333	206	0.6186	327	0.4895	0.5465	0.4242	0.7713	0.5720
0.4	EW	336	151	0.4494	254	0.3802	0.4119	0.6256	0.6393	0.6324
	RM	388	242	0.6237	339	0.5075	0.5596	0.4466	0.7557	0.5809
0.5	EW	405	188	0.4642	269	0.4027	0.4313	0.6229	0.6387	0.6307
	RM	491	308	0.6273	350	0.5240	0.5710	0.4649	0.7317	0.5832
0.6	EW	475	221	0.4653	279	0.4177	0.4402	0.6197	0.6328	0.6262
	RM	624	393	0.6298	353	0.5284	0.5747	0.4779	0.7335	0.5920
0.7	EW	568	261	0.4595	285	0.4266	0.4425	0.6201	0.6353	0.6277
	RM	755	493	0.6530	359	0.5374	0.5896	0.4908	0.7370	0.6014
0.8	EW	674	298	0.4421	288	0.4311	**0.4366**	0.6203	0.6362	0.6282
	RM	880	570	0.6477	363	0.5434	**0.5910**	0.4938	0.7363	0.6030
0.9	EW	819	348	0.4249	288	0.4311	0.4280	0.6195	0.6395	0.6294
	RM	945	584	0.6180	363	0.5434	0.5783	0.4942	0.7350	0.6027

Ncp denotes the number of the correct predictions that match at least a true complex and Ncb denotes the number of the true complexes that match at least one predicted complex.

### Performance comparison with other methods

The performance comparison with the-state-of-art unsupervised methods including MCODE, COACH, CMC and ClusterONE is shown in table 8. In order to compare with these methods as fair as possible, we designed an experiment method similar to the five-fold-cross-validation to obtain the complexes with our method: the 668 positive examples are divided into five folds {S_1_, S_2_, S_3_, S_4_, S_5_}. For each cross validation experiment, four folds (plus 422 intermediate samples and 2004 negative samples) are used as the training set and then the trained Regression model is used to detect the complexes in the DIP PIN. Since the detected complexes may include the ones in the training set, the predicted complexes matched with the training set are removed (the match threshold is set to 0.9 calculated by Equation (6)). For example, if the training set is {S_1 _U S_2 _U S_3 _U S_4_}, then the set of remained complexes is supposed to include the complexes in S_5_.

**Table 8:** Performance comparison with MCODE, COACH, CMC and ClusterONE on four PIN datasets

Dataset	Method	Num	P	R	F	Sn	PPV	Acc
DIP	MCODE	79	0.5570	0.1332	0.2150	0.2758	0.6880	0.4356
	COACH	747	0.4351	0.5195	0.4735	0.4779	0.6921	0.5751
	CMC	262	0.5687	0.4102	0.4766	0.4791	0.7241	0.5890
	ClusterONE	354	0.5113	0.4072	0.4533	0.3903	0.7124	0.5273
	Ours	613	0.6232	0.5269	0.5710	0.4764	0.7375	0.5927
Gavin	MCODE	78	0.8718	0.2809	0.4249	0.4174	0.7017	0.5412
	COACH	326	0.7393	0.6086	0.6676	0.6277	0.7162	0.6705
	CMC	202	0.7228	0.4176	0.5294	0.3817	0.7067	0.5194
	ClusterONE	200	0.8050	0.5693	0.6669	0.6211	0.7048	0.6617
	Ours	275	0.8145	0.5730	0.6728	0.5083	0.7526	0.6185
Krogan	MCODE	63	0.6349	0.1544	0.2484	0.4439	0.4865	0.4642
	COACH	570	0.4439	0.4865	0.4642	0.502	0.6575	0.5745
	CMC	242	0.5909	0.3555	0.4439	0.3263	0.7215	0.4852
	ClusterONE	258	0.5349	0.4381	0.4817	0.4865	0.7567	0.6067
	Ours	465	0.5591	0.4955	0.5254	0.4944	0.7189	0.5962
Collins	MCODE	111	0.8468	0.431	0.5713	0.5438	0.7600	0.6429
	COACH	251	0.6972	0.5651	0.6243	0.6275	0.7931	0.7054
	CMC	172	0.6919	0.4234	0.5253	0.4882	0.7336	0.5985
	ClusterONE	180	0.8222	0.5958	0.6909	0.6526	0.7275	0.6891
	Ours	150	0.8133	0.5096	0.6266	0.6338	0.7431	0.6863

After five round such experiments are performed, the five result sets of the remained complexes are combined which is supposed to include the complexes in {S_1 _U S_2 _U S_3 _U S_4 _U S_5_}, and then the similar complexes with the match score higher than 0.6 (which is determined through our experiments and can achieve the best performance) are removed. Finally, the remained detected complexes (which are achieved avoiding the problem that the training and testing set overlap) are used as our final result, which is then evaluated with {S_1 _U S_2 _U S_3 _U S_4 _U S_5_} (the 668 positive examples). In this way we avoid the problem that the training and testing set may overlap. Since MCODE, COACH, CMC and ClusterONE are unsupervised methods, their results are directly obtained on the PIN, and their optimal parameters are used.

The experiments were performed on four PIN datasets: DIP, Gavin [30], Krogan [31] and Collins [32] (their details are shown in Table 9). In these networks, interactions with GO similarities less than 0.9 are regarded as false positive interactions and deleted as described in previous section.

**Table 9:** Details of four PIN datasets

Dataset	#original proteins	#original interactions	#remained proteins	#remained interactions
DIP	4928	17201	3449	11081
Gavin	1430	6531	1304	5941
Krogan	3581	14076	2270	9218
Collins	1622	9074	1513	8949

As shown in Table 8 on the DIP dataset, the widely used dataset in complex detection field, our method obtains the best result on almost every evaluation metric on the DIP dataset. In term of the F-measure, the most frequently used evaluation metric, our method achieves the highest performance (0.5710), which is much superior to those of MCODE (0.2150), COACH (0.4735), CMC (0.4766) and ClusterONE (0.4533). The performances (measured with F-measure) of our method are also best on other PIN datasets except Collins (on Collins dataset our method's performance (0.6266) is inferior to that of ClusterONE (0.6909), but still better than others).

The main advantage of our method over other methods is that it uses the supervised learning method in the complex detection process, which makes full use of the information of available known complexes to achieve better performance.

Qi et al. are the first to import the supervised learning-based method into the complex detection. Table 10 gives the performance comparison between their method and ours. Since the program used by Qi et al. is not available, we use their published results [15]. Qi et al. used MIPS and TAP06 as the positive sets. Thus, in order to make the results as comparable as possible, our datasets were processed in the same way as Qi et al. did, i.e. the complexes composed of a single protein or a pair of proteins were filtered out. After the filter processing, 200 complexes in MIPS remained and 150 complexes in TAP06 remained. It should be pointed out that the number of remaining complexes in TAP06 in Qi et al.'s and our method are almost the same (152 to 150), whereas those remaining in MIPS were markedly different (101 to 200). Moreover, in line with Qi et al.' method, we only kept the proteins from the two true complex sets in the PIN, yielding 1353 proteins and 5072 interactions. We conducted experiments using MIPS as the positive training set and TAP06 as the testing set and vice versa.

**Table 10:** Performance comparison with Qi et al.'s method

	MIPS(training set)						TAP06(training set)					
	
	TAP06(testing set)						MIPS(testing set)					
**Method**	**Num**	**Ncp**	**P**	**Ncb**	**R**	**F**	**Num**	**Ncp**	**P**	**Ncb**	**R**	**F**
Ours	271	115	0.424	65	0.433	**0.429**	262	128	0.489	105	0.525	**0.506**
SCI-BN			0.312		0.489	0.381			0.219		0.537	0.312
SCI-SVM			0.247		0.377	0.298			0.176		0.379	0.24
MCODE	45	19	0.422	19	0.127	0.195	45	18	0.4	20	01	0.160
ClusterOne	173	83	0.480	69	0.46	**0.470**	173	74	0.428	87	0.435	0.431
COACH	294	114	0.387	80	0.533	0.449	294	107	0.364	99	0.495	0.419
CMC	161	72	0.447	53	0.353	0.395	161	74	0.460	76	0.380	0.416
	**MIPS, Aloy and SGD(training set)**						**TAP06, Aloy and SGD(training set)**					
	**TAP06(testing set)**						**MIPS(testing set)**					
Ours1	274	119	0.434	69	0.46	**0.447**	270	142	0.526	102	0.51	**0.518**

In our experiments, we set the clique_size ≥ 3 and merg_thred 0.8, with all the evaluation metrics in Table 10 computed the same way as in Qi et al.' method. Here, it should be pointed out that in the Qi et al.' method, the measure that defines the predicted complex matching the true complex is different from the NA (A, B) value computed in Equation (6). Qi et al. assumed that, if the common proteins both in the predicted complex and the true complex constitute more than 50% of each one, the predicted complex is taken as a match to the true complex. The precision, recall and F-measure are all calculated based on this definition and are shown in Table 10.

As can be seen from Table 10 on both training-testing sets, the F-measures of our method are better than those of Qi et al.'s method. Especially when TAP06 is used as the training set and MIPS as the testing set, our F-measure is 19.4 percentage points higher than that of Qi et al.'s method (0.312 to 0.506). Although the results are not fully comparable for different numbers of remaining complexes in MIPS, they still show the effectiveness of our method.

We also compared our method with that of the Shi et al. (a semi-supervised prediction model with neural network. They used MIPS as both the training and testing set and achieved a performance of 0.397 in F-measure (0.333 in precision and 0.491 in recall) on DIP database. With the same experimental setting and evaluation metrics, our method obtains a better performance of 0.5144 in F-measure (0.4194 in Precision and 0.665 in Recall).

Better performance of our method over other two supervised learning based methods, Qi et al. and Shi et al. may be due to the following three key reasons: (1) Firstly, as discussed in previous section, our method introduces some new features from the weighted network: the density, mean and maximum degrees of the weighted network, which prove to be quite effective for the performance improvement. Secondly, in our method, the initial cliques used are the maximal cliques found by the Cliques algorithm and has been proven to be more effective than expanding from the seeding proteins. In contrast, in the other two methods, each seeding protein is connected to its highest weight neighbor and the pair is subsequently used as the starting cliques. We conducted an experiment in which the starting cliques were selected with such method and other experimental setting unchanged and an F-measure of 0.5418 was achieved, which is inferior to the result of our method (0.5910). Finally, our method introduces the three categories training set for the first time. Since the more samples and additional categories provide more information for the regression model training, the learned model becomes more discriminative.

For comparison purpose, the performances of MCODE, COACH, CMC and ClusterOne on the same PIN are also presented in Table 10. On the testing set of MIPS, our method also outperforms others. However, on the testing set of TAP06, the performances of COACH and ClusterOne are better than that of our method. The reason is the limited size of positive samples (200 complexes from MIPS). When we introduced more positive samples from Aloy and SGD (total 263 complexes from MIPS, Aloy and SGD), a much better performance is achieved (0.447 in F-measure, the last row in Table 10 denoted as "Ours1") which is very close to those of COACH and ClusterOne (0.449 and 0.470 in F-measure, respectively). Similarly, on the testing set of MIPS, when more positive samples are introduced, a better performance is achieved (improved from 0.506 to 0.518 in F-measure). This shows that, if more training samples are provided, as a supervised learning method our method can achieve better performance than the unsupervised methods.

### Statistical evaluation of the predicted protein complexes

To substantiate the biological significance of our predicted complexes, we calculated their function p-values, which represent the probability of co-occurrence of proteins with common functions. As such, low p-value of a predicted complex generally indicates that the collective occurrence of these proteins in the complex does not occur merely by chance and thus the complex has high statistical significance.

**Figure 4:**
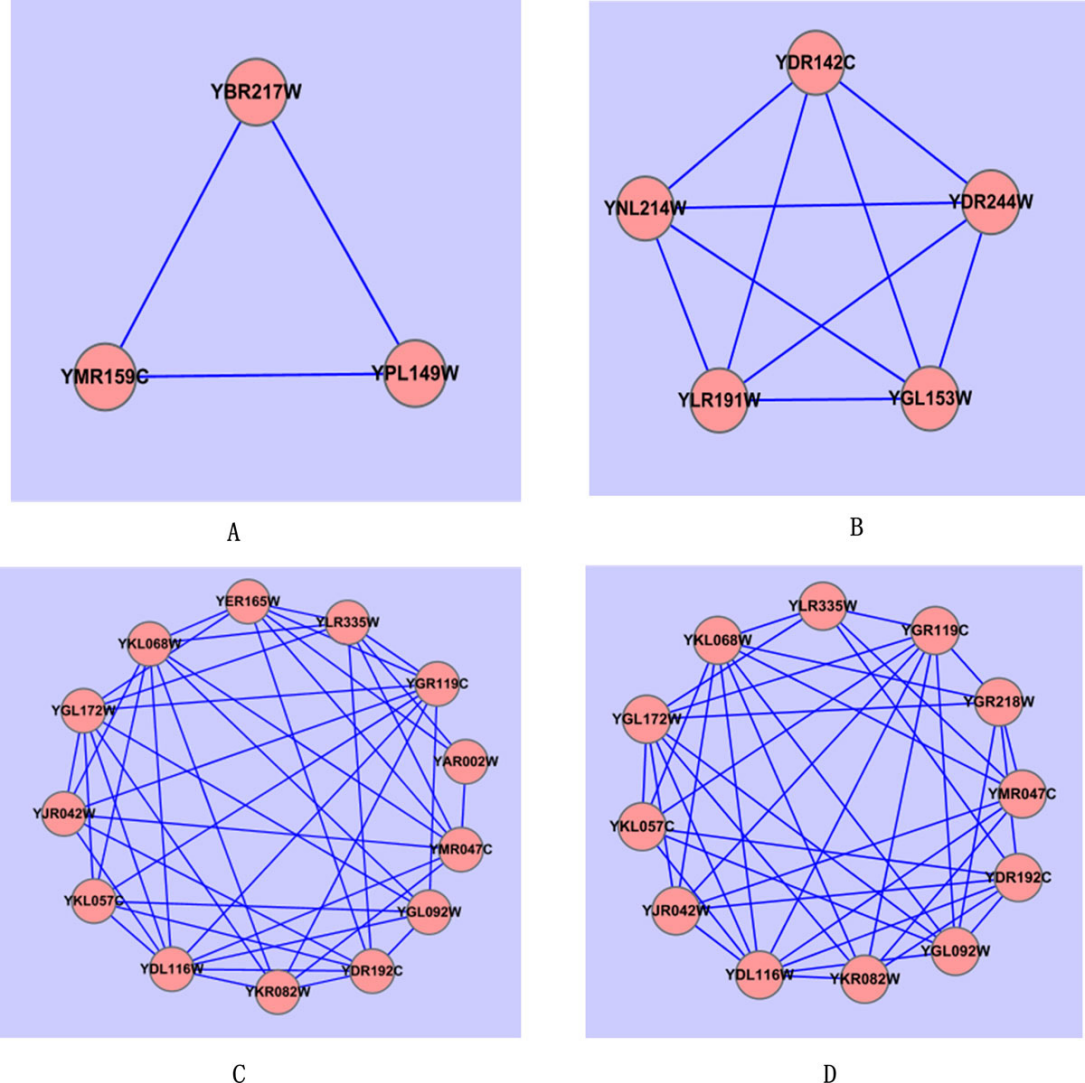
**Four complexes which don't match the true complexes**.

Table 11 gives ten examples of the low p-value predicted complexes that are matched with the true complexes from the benchmark (In our experiments, the p-values of complexes are calculated with the SGD's GO::TermFinder [33]. However, although some of our predicted complexes with low p-values were not matched with the true complexes, they still have high biological significance, as some of them may be true complexes that are still undiscovered. Examples of such complexes are given in Table 12 and Figure 4 and might be of use for biologists looking for new protein complexes.

**Table 11:** Ten predicted complexes with low p-values that match the true complexes

ID	Complex	Match_score	p-value		
			
			GO_Process	GO_Function	GO_Component
1	YLR148W YMR231W YLR396C YPL045W YDR080W	0.83	6.72e-14	2.90e-10	1.10e-15
2	YMR224C YNL250W YDR369C	1.0	2.90e-07	3.58e-10	2.93e-10
3	YLR438C-A YNL147W YBL026W YMR268C YER112W YDR378C YJR022W YER146W	1.0	1.57e-13	1.10e-07	1.51e-24
4	YPR162C YJL194W YLL004W YNL261W YBR060C YHR118C YML065W	0.86	3.38e-17	2.43e-16	1.15e-18
5	YDL225W YLR314C YHR033W YCR002C YJR076C YHR107C	0.83	4.76e-07	1.73e-10	2.39e-13
6	YMR047C YLR335W YLR347C YAR002W YNL189W	0.8	2.51e-09	4.23e-06	1.92e-03
7	YBL016W YDR103W YLR362W YDL159W YGR040W	1.0	1.21e-10	6.83e-06	4.60e-04
8	YML056C YIL079C YJL050W YOL115W YDL175C	0.8	5.18e-11	1.62e-08	3.50e-11
9	YPL083C YMR059W YAR008W YLR105C	1.0	2.27e-13	9.09e-14	2.54e-13
10	YKL166C YJL164C YPL203W YIL033C	1.0	3.70e-08	5.21e-10	8.47e-10

**Table 12:** Four predicted complexes with low p-values that don't match the true complexes

ID	Complex	Match_score	p-value		
			
			GO_Process	GO_Function	GO_Component
A	YPL149W YBR217W YMR159C	0.0	2.41e-08	1.95e-10	2.28e-10
B	YNL214W YDR244W YLR191W YDR142C YGL153W	0.0	2.92e-16	9.30e-03	1.49e-09
C	YMR047C YJR042W YKL068W YDR192C YDL116W YGR119C YKL057C YKR082W YLR335W YGL092W YER165W YGL172W YAR002W	0.21	1.57e-23	8.46e-26	2.92e-06
D	YMR047C YJR042W YKL068W YGR218W YDR192C YDL116W YGR119C YLR335W YKR082W YKL057C YGL092W YGL172W	0.22	3.39e-18	1.78e-23	1.65e-09

## Conclusions

Protein complexes are important for understanding principles of cellular organization and function. Since high-throughput experimental techniques produce a large amount of protein interactions, many complex detection algorithms have been proposed. However, most of the current methods are only based on the topological structure of the PIN and do not make use of the information of the available known complexes.

In this paper, we present a supervised learning-based method to detect complexes from PIN. In this method, through constructing a training set, a Regression model is obtained that is subsequently used to assess the detected complexes for the cliques filtering, growth, and candidate complex filtering. The evaluation and analysis of our predictions demonstrate the several advantages of our method over other state-of-the-art techniques. Firstly, our method is a supervised learning-based method that can make full use of the information of the available known complexes instead of being only based on the topological structure of the PIN. That also means, if more training samples are provided, our method can achieve better performance than those unsupervised methods. Secondly, we design the rich feature set to describe the properties of the known complexes, which includes not only the features from the unweighted network, but also those from the weighted network built based on the Gene Ontology information. The weighted network features achieve a much better performance than the unweighted network features, which proves the effectiveness of the usage of Gene Ontology. Thirdly, our Regression model utilizes additional "uncertainty" samples and, therefore, becomes more discriminative, whose effectiveness for the complex detection is clearly indicated by our experimental results.

Our future work will focus on exploring more effective features for the complex detection in PIN. Especially, extracting the features from the biomedical resources such as Gene Ontology may be a promising approach. In addition, cooperation with biomedical expert on protein complex detection in some certain disease PIN will also be one of our next step works through which the effectiveness of our method can be further verified.

## Competing interests

The authors have declared that no competing interests exist.

## Authors' contributions

ZHY and NT conceived of the study, carried out its design and drafted the manuscript. FYY and NT performed the experiments. FYY, HFL, JW and ZWY participated in its design and coordination, and helped to draft the manuscript. All authors read and approved the final manuscript.

## Declarations

Publication of this article was funded by the following grants: the Natural Science Foundation of China (grant no. 61070098, 61272373 and 61340020), Trans-Century Training Programme Foundation for the Talents by the Ministry of Education of China (grant no. NCET-13-0084) and the Fundamental Research Funds for the Central Universities (grant no. DUT13JB09 and DUT14YQ213).

This article has been published as part of *BMC Systems Biology *Volume 8 Supplement 3, 2014: IEEE International Conference on Bioinformatics and Biomedicine (BIBM 2013): Systems Biology Approaches to Biomedicine. The full contents of the supplement are available online at http://www.biomedcentral.com/bmcsystbiol/supplements/8/S3.
